# Myasthenia Gravis for Spine Surgery: An Ardous Anaesthetic Journey

**DOI:** 10.7759/cureus.50695

**Published:** 2023-12-17

**Authors:** Amlan Swain, Seelora Sahu, Merina Sam, Deb Sanjay Nag

**Affiliations:** 1 Department of Anaesthesiology, Tata Main Hospital, Jamshedpur, IND

**Keywords:** muscle relaxation, prone position surgery, lumbar surgery, anaesthesia, myasthenia gravis

## Abstract

Myasthenia gravis (MG) is a neurological disorder involving the post-synaptic neuromuscular junction and is caused by the autoimmune destruction of acetylcholine receptors with ensuing muscular weakness. Rarely is the disease process in MG compounded with other comorbidities and distinctive surgical challenges, such as the prone position in spine surgery, presenting unique challenges in the anesthetic management of such cases. This case series and the ensuing discussion describe the successful perioperative management of two cases of MG undergoing neuro-surgical management for lumbar spine pathologies.

## Introduction

Myasthenia gravis is an autoimmune disorder that specifically targets and reduces the availability of nicotinic acetylcholine receptors at the postsynaptic neuromuscular junction. It is characterized by a fatigable weakness of skeletal muscles that improves on rest and is associated with disorders such as hypothyroidism, rheumatoid arthritis, systemic lupus erythematosus, and diabetes mellitus. Anesthetic management of cases of MG poses unique challenges due to the pathological factors involved and their interplay with medications used in administering anesthesia [1]. Peri-operative management of spine surgery in itself is challenging, and it adds up more when a patient has a case of MG.

## Case presentation

Case 1

**Figure 1:**
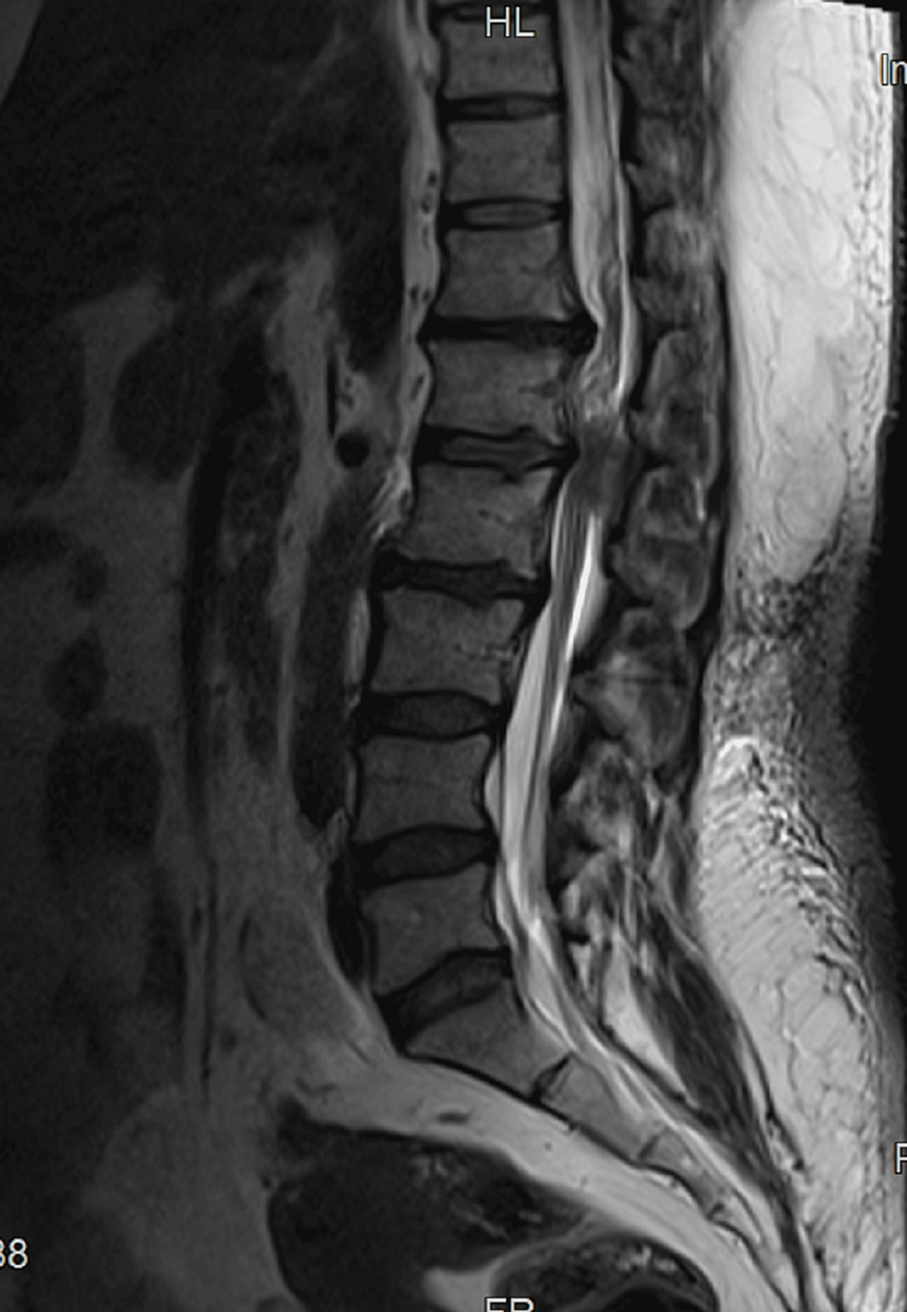
Sagittal section of the spine showing prolapsed intervertebral disc (PIVD) at D12- L1 and L1-L2 spinal levels D: Dorsal vertebra, L: Lumbar vertebra

A 40-year-old female was a known case of hypertension, diabetes mellitus, dyslipidemia, and MG (Class IIa), diagnosed two years ago. Following an episode of myasthenic crisis, she was tracheostomized and still had the tracheostomy tube in situ. Presently, she was scheduled for an elective dorsal decompressive laminectomy in view of severe neurological symptoms of back pain and radiculopathy due to prolapsed intervertebral disc (PIVD) at D12-L1 and L1-L2 intervertebral levels (Figure 1).

**Figure 2:**
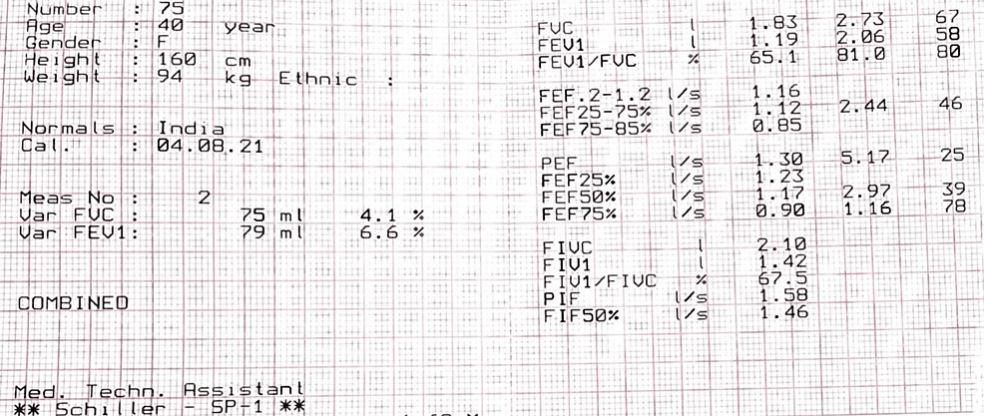
The pulmonary function tests of case 1 FVC: Forced vital capacity, FEV1: Forced expiratory volume, FEF: Forced expiratory flow, PEF: Peak expiratory flow, PIF: Peak inspiratory flow, FIF: Forced inspiratory flow, FIV: Forced inspiratory volume, FIVC: Forced inspiratory vital capacity

She was a morbidly obese patient with cushingoid features and was on multiple medications (Pyridostigmine, Azathioprene, Atorvastatin, Gabapentin, Tizanidine, Methylcobalamine, Bilastine, Metformin, Teneligliptine, Prednisolone, and Hydroxyzine) for her comorbidities. She was mostly bedridden due to her muscle weakness secondary to MG and, more recently, due to back pain and radiculopathy as a result of the PIVD. Her systemic examination and blood investigations were otherwise unremarkable, and a preoperative X-ray of the neck revealed a tracheostomy tube in situ without any obvious compression of the trachea. Pulmonary function tests (PFT) done with tracheostomy stoma closure showed fixed extra-thoracic obstruction and severe restrictive lung disease (Figure 2).

Following consultation with multiple specialists, including neurology, endocrinology, and neurosurgery, the patient underwent five cycles of plasmapheresis before surgery, along with steroid supplementation to ameliorate any chances of precipitating a myasthenic crisis and improve neuromuscular function. A right femoral central venous access was secured preoperatively. The patient was taken up for surgery under American Society of Anesthesiologists (ASA) physical status III, and the surgery was conducted under general anesthesia (GA) with positive pressure ventilation (PPV). Standard ASA monitors were used during the surgery. The anesthesia regimen included standard anesthetic drugs like fentanyl, propofol, sevoflurane, and low doses of atracurium. Perioperative steroid cover was administered in the form of intravenous hydrocortisone 100 mg after induction of anesthesia, followed by an infusion at a rate of 10 mg/hour. The patient was made comfortable with proper cushioning of pressure points. The intraoperative course was uneventful, and during surgery, the atracurium requirement was minimal (30 mg at induction and four intermittent boluses of 5 mg). After surgery, the patient was turned to a supine position. A neuromuscular blocker (NMB) reversal agent was not administered, and she was closely monitored till she was able to open her eyes and was generating adequate tidal volume after a few minutes. Subsequently, she was shifted to the surgical step-down care unit (surgical ICU) for continuous monitoring on the T-piece and spontaneous ventilation for 48 hours. She was later discharged home after four days on T-piece while maintaining oxygen saturation in room air.

Case 2

**Figure 3:**
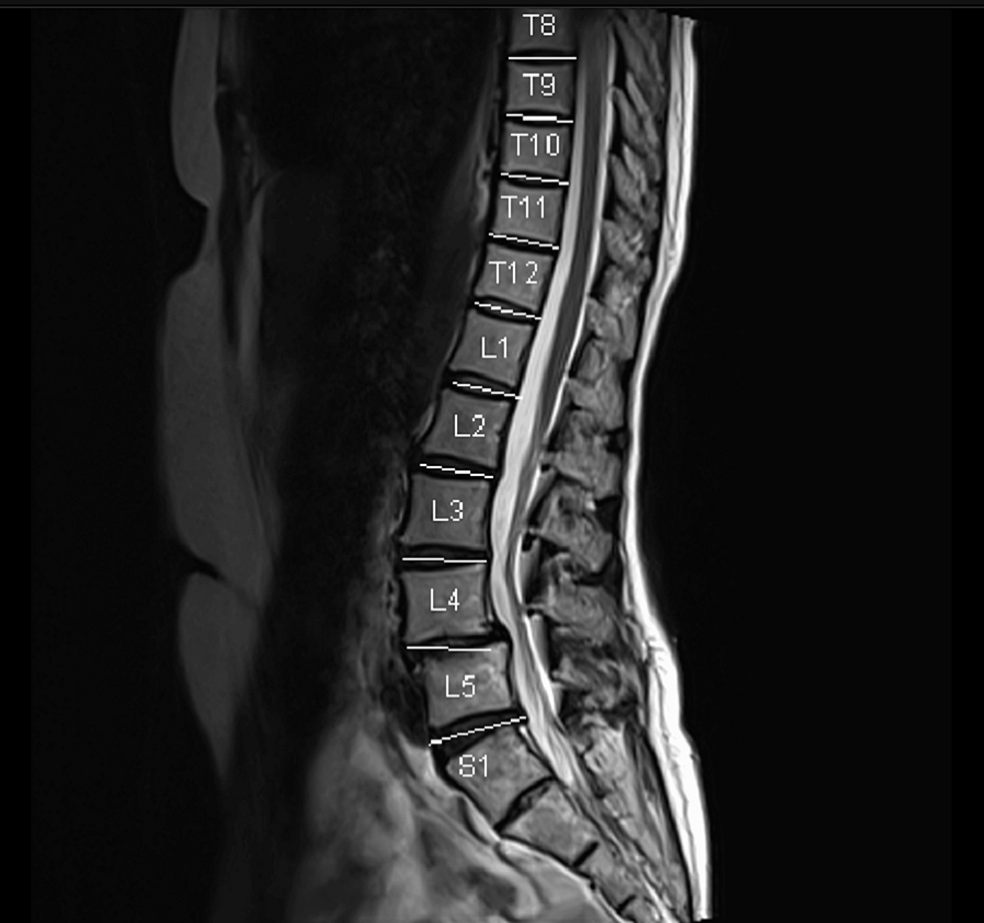
Sagittal section of spine showing spondylolisthesis at the level of L4-L5 intervertebral space. T: Thoracic vertebra, L: Lumbar vertebra, S: Sacral vertebra

A 53-year-old female was a known case of MG (Class IIa) and hypothyroidism (post-ablation of Grave’s diseases) for five years, inflammatory polymyositis, and was on multiple medications for the same (Pyridostigmine, Duloxetine, Mycophenolate, Gabapentin, and Thyroxine [100 mcg]). She was posted for posterior lumbar interbody fusion (PLIF) in view of spondylolisthesis at the L4-L5 intervertebral level (Figure 3).

**Figure 4:**
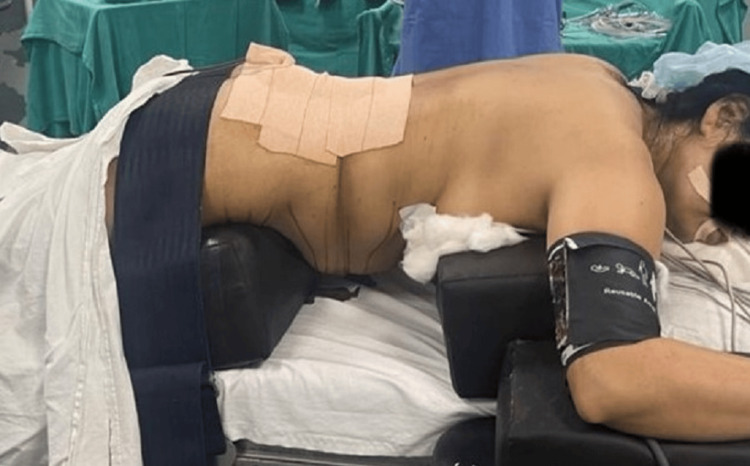
Patient in prone position

On preoperative evaluation, there was no history of respiratory symptoms in the recent past, and her effort tolerance was acceptable (metabolic equivalent of tasks > 4). Routine investigations and thyroid profile were within the normal range, the ECG showed sinus bradycardia, and a 2D echocardiogram was done within normal limits. This patient was also taken up for surgery under ASA grade III, and she received GA with PPV and endotracheal intubation. The anesthetic regimen included fentanyl, etomidate, sevoflurane, and a minimal dose of atracurium (20 mg at induction and two intermittent boluses of 5 mg). The surgery was performed in the prone position with adequate cushioning of pressure points (Figure 4).

At the end of the surgery, she was turned back to the supine position. When she was spontaneously opening her eyes and generating an adequate tidal volume, she was extubated without the administration of a NMB reversal agent, similar to the first case. She was shifted to the ICU for continuous monitoring for 24 hours, where the postoperative course was uneventful, and was subsequently discharged home after two days.

## Discussion

We report the successful management of two challenging cases of MG with multiple comorbidities posted for neurosurgery of the spine under GA. The administration of anesthesia to our patients posed special challenges due to the inherent pathogenic muscular weakness of the patient undergoing spine surgery in the prone position, compounded by the need for muscle paralysis to provide optimum and safe surgical conditions.

The anesthetic concerns in our first patient were multiple, including MG, obesity, cushingoid features, a bedridden patient with a tracheostomy tube in situ for the past two years, severe restrictive lung involvement, and difficult intravenous access. There was also the challenge of proper positioning for surgery and preventing airway complications (dislodgement of the tracheostomy tube or airway obstruction). She required plasmapheresis preoperatively (1 and 2 days prior to surgery) to stabilize the neurological condition and prevent myasthenic crises in the perioperative period. Plasmapheresis is known to improve perioperative outcomes by attenuating the dependence on ventilatory support and preventing myasthenic crises [2,3]. The second case, on the other hand, had hypothyroidism (post-ablation of Grave’s disease five years ago) and inflammatory polymyositis in addition to MG. Preoperative optimization of underlying co-morbid conditions and continuation of the medications for MG were crucial to the successful management of both cases. The preoperative PFT aided in the assessment of respiratory insufficiency and planning for intraoperative and postoperative ventilatory management [4].

Peripheral nerve blocks (PNB) and regional anesthesia (RA) are the preferred techniques of anesthetic management in patients with MG, as these approaches avoid the respiratory complications (respiratory failure) of GA [1]. However, the nature and site (lumbar spine) of surgery in our patients eliminated any possibility of using nerve blocks or RA. In surgeries where GA is deemed inevitable, balanced anesthesia using either intravenous-inhalational or only intravenous anesthesia is the advocated technique [1,5]. We did the same in both of our cases.

One of the main concerns in patients with MG who are administered GA is related to the use of muscle relaxants, wherein the inherent muscle weakness can be compounded by both depolarizing and non-depolarizing muscle relaxants, causing difficulty in recovery from muscle paralysis and resumption of optimum muscular tone and respiratory functions [6-8]. To circumvent this concern, numerous studies have been carried out reporting that surgeries have been done under GA without using muscle relaxants [8,9]. Our case series is unique because of the administration of GA in a rare combination of patients with MG requiring spine surgery. Spine surgery involves neural structures, and even the slightest movement during surgery can predispose the patient to catastrophic neurological sequelae. Hence, we decided to use muscle relaxants in significantly lower than normal dosages to provide optimal and safe operating conditions. We avoided succinylcholine in our patients to avoid prolonged blockade, as both of them were on cholinesterase inhibitor therapy [10]. We used lower doses of sevoflurane and propofol due to their desirable and proven pharmacological properties of rapid clearance, which were of benefit to these patients [11]. It is an established fact that reversal of neuromuscular blockade using Neostigmine is unpredictable in patients with MG, especially if they are taking anticholinesterase medication, which has been known to precipitate a cholinergic crisis. To avoid these complications, Atracurium was chosen as the non-depolarizing neuromuscular blocking agent because of its alternate pathway of metabolism and elimination (Hoffman degradation), which reduces its dependence on Neostigmine for the reversal of muscle paralysis [12,13]. At the end of surgery, both our patients were allowed to recover spontaneous respiratory effort and muscle tone by themselves before putting them on a T-piece (Case 1) or extubating (Case 2). Criteria favoring successful extubation, which is challenging in MG patients, include a normal level of consciousness, a respiratory rate less than 30/min, and an adequate tidal volume of at least 5 ml/kg body weight. These criteria were met by both of our patients [14]. Both of our patients were closely monitored clinically for postoperative residual muscle paralysis and cardiorespiratory complications in the postoperative recovery area.

The risk of precipitating myasthenic crisis and cholinergic crisis, especially in stressful situations (like surgery), was mitigated by avoiding use of any sedative or depressant medications in the pre-operative period, continuing the medications of MG throughout the perioperative tenure, and avoiding administration of anticholinesterase neuromuscular block reversal agents at the end of surgery [2,15].

## Conclusions

A multidisciplinary approach, robust preoperative preparation, meticulous intraoperative management, and vigilant post-operative care go a long way in negotiating a tumultuous peri-operative course in patients with MG for spine surgery.
